# Identification of BRCA1 As a Potential Biomarker for Insulin-Like Growth Factor-1 Receptor Targeted Therapy in Breast Cancer

**DOI:** 10.3389/fendo.2017.00148

**Published:** 2017-06-29

**Authors:** Tali Cohen-Sinai, Zoya Cohen, Haim Werner, Raanan Berger

**Affiliations:** ^1^Department of Human Molecular Genetics and Biochemistry, Sackler School of Medicine, Tel Aviv University, Tel Aviv, Israel; ^2^Institute of Oncology, Chaim Sheba Medical Center, Tel Hashomer, Israel; ^3^Yoran Institute for Human Genome Research, Tel Aviv University, Tel Aviv, Israel

**Keywords:** insulin-like growth factor-1, insulin-like growth factor-1 receptor, BRCA1, targeted therapy, breast cancer

## Abstract

The insulin-like growth factor-1 receptor (IGF1R) emerged in recent years as a promising therapeutic target in oncology. Identification of potential biomarkers capable of predicting response to IGF1R-targeted therapy is of cardinal importance. Tumor suppressor BRCA1 has important roles in multiple pathways, including gene transcription, DNA damage repair, and control of apoptosis. Early studies have identified the *IGF1R* gene as a downstream target for inhibitory regulation by wild-type, but not mutant, BRCA1. The aim of the present study was to evaluate the hypothesis that the mutational status of BRCA1 may influence the ability of IGF1R-directed therapies to efficiently inhibit the IGF1R axis. Using breast cancer-derived cell lines expressing a wild-type or a mutant BRCA1, we demonstrate that the capacity of MK-0646, a monoclonal antibody antagonist to the human IGF1R, to inhibit insulin-like growth factor-1-stimulated IGF1R and downstream mediators’ phosphorylation was impaired in mutant BRCA1-expressing cell lines. In addition, the antibody was able to reduce proliferation of wild-type BRCA1-expressing cells but had a reduced inhibitory effect in mutant BRCA1-expressing cells. In summary, our data indicate that the mutational status of BRCA1 must be taken into account when selecting patients for IGF1R targeting protocols.

## Introduction

The proliferation of breast epithelial cells depends on the concerted actions of a number of steroid hormones and peptide growth factors. The insulin-like growth factor system consists of a network of circulating ligands [insulin-like growth factor-1 (IGF1), IGF2], cell-surface receptors, and IGF-binding proteins (IGFBPs) that are involved in multiple physiological and pathological processes (1, 2). The IGF system has an important role in development and maturation of the mammary gland as well as in breast cancer initiation and progression (3–5). The IGF1 receptor (IGF1R), which mediates the biological actions of both ligands, exhibits potent antiapoptotic and cell-survival activities and is regarded as a major player in breast cancer development. The IGF1R is highly expressed in many malignant cells whereas, on the other hand, cells with a targeted disruption of the IGF1R gene, for the most part, do not undergo transformation (6–9). Most basic, clinical and epidemiological studies agree with the notion that constitutive activation of the IGF1R tyrosine kinase domain constitutes a relatively common event in cancer cells (10, 11). The prognostic significance of IGF1R levels and activation status, however, remains controversial. Analysis of the predictive impact of IGF1R expression among patients with early breast cancer and among breast cancer subtypes revealed that IGF1R levels correlated with good prognostic markers (7). Furthermore, IGF1R was shown to be differentially expressed with variable prognostic impact among breast cancer subtypes. In recent years, the IGF1R emerged as a promising therapeutic target in breast and other types of cancer. However, results of Phase I/II clinical trials have shown variable responses to IGF1R-directed therapies (12–14). Therefore, identification of biomarkers that can predict response to targeted therapy is a major goal in cancer treatment.

The breast and ovarian cancer susceptibility gene (BRCA1) is a tumor suppressor whose mutation was correlated with the appearance of familial breast and/or ovarian cancer at young ages (15–17). Transcription factor BRCA1 participates in multiple biological pathways, including DNA damage repair, apoptosis, and transcription (18, 19). Comprehensive analyses conducted in our laboratory have identified the *IGF1R* gene as a downstream target for BRCA1 action (20–22). In agreement with its tumor suppressor role, exogenous BRCA1 expression in breast cancer cells led to reductions in endogenous IGF1R protein and mRNA levels and a marked decrease in *IGF1R* promoter activity. On the other hand, a mutated *BRCA1* gene encoding a truncated version of the molecule (185delAG) had no effect on *IGF1R* gene expression. A bidirectional link between the IGF1 and BRCA1 signaling pathways was suggested by studies showing that cellular levels of BRCA1 are upregulated by ambient concentrations of IGF1 (23). In addition, immunohistochemical analyses of IGF1R levels in a collection of primary breast tumors derived from *BRCA1* mutation carriers and non-carriers revealed a higher score in BRCA1-associated tumors compared to sporadic tumors (24). Non-tumorous breast tissue of 185delAG BRCA1 mutation carriers had a higher IGF1R score than tissue derived from non-carriers. These results are consistent with the notion that loss of inhibitory control as a result of *BRCA1* mutation may lead to enhanced IGF1R expression and, eventually, increased cancer incidence.

Given the physical and functional interactions between the BRCA1 and IGF1 signaling pathways, and to expand our previous studies on the transcriptional regulation of the *IGF1R* gene by BRCA1, we evaluated in the present study the impact of BRCA1 mutations on the ability to target the IGF1R in breast cancer cells. Using a specific IGF1R monoclonal antibody we demonstrate that (1) the ability of the targeting agent to inhibit the IGF1 signaling pathway was impaired in mutant BRCA1-expressing cells; (2) the effect of the blocking antibody on inhibition of IGF1-mediated proliferation was diminished in mutant BRCA1 cells; and (3) the synergistic effect of anti-IGF1R therapy along with chemotherapy was reduced in mutant BRCA1 cells. We conclude that assessment of BRCA1 mutational status might be of importance in selecting patients for future IGF1R-directed clinical interventions.

## Materials and Methods

### Cell Lines

The following breast cancer cell lines were employed in the present study: MCF7, MCF10A, HB2, MDA-MB-231, and HCC1937. The MCF7 cell line (ER+, PR+) is an aggressive adenocarcinoma line derived from a metastatic site. The MCF10A cell line (ER−, PR−) is a non-tumorigenic, telomerase-immortalized breast epithelial cell line. The HB2 cell line was originated by introduction of the SV40 large T antigen into MTSV mammary luminal epithelial cells. HB2 is usually regarded as a non-neoplastic breast line (25). MDA-MB-231 (ER−, PR−) is a breast cancer cell line derived from a pleural effusion. MCF7, MCF10A, HB2, and MDA-MB-231 cell lines were obtained from the American Type Culture Collection, Manassas, VA, USA. All four cell lines express a wild-type BRCA1 (26). The HCC1937 cell line was derived from a primary ductal carcinoma. Mutational analysis identified a homozygous BRCA1 5382C mutation in this cell line. HCC1937 cells were kindly provided by Dr. L. C. Brody (National Human Genome Research Institute, Bethesda, MD, USA). MCF7 and HCC1937 cells were maintained in high glucose DMEM supplemented with 10% fetal bovine serum (FBS), 2 mM l-glutamine, and antibiotics. MCF10A cells were maintained in DMEM F12 medium supplemented with 5% horse serum, 2 ng/ml epidermal growth factor, 100 ng/ml cholera toxin, 50 ng/ml hydrocortisone, and 10 μg/ml insulin. HB2 and MDA-MB-231 cells were maintained in high glucose DMEM supplemented with 10% FBS, 2 mM l-glutamine, 5 μg/ml hydrocortisone, and 10 μg/ml insulin. All cells were propagated in a 37°C humidified incubator with 5% CO_2_.

### IGF1R Inhibitor

MK-0646 (*Dalotuzumab*; Merck, Sharp and Dohme Ltd., Whitehouse Station, NJ, USA) is a humanized antibody antagonist to the human IGF1R. MK-0646 was diluted in 20 mM histidine and 150 mM NaCl and used at a concentration of 10 μg/ml.

### Transient Transfections and Viral Infections

To generate wild-type BRCA1-expressing HCC1937 cells, naïve HCC1937 cells were transiently transfected with 10 μg of a pcDNA3-BRCA1 expression vector, or empty pcDNA3 vector (Invitrogen, Carlsbad, CA, USA), using the jetPRIME^®^ reagent (Polyplus Transfection, Illkirch, France). The expression vector was constructed by cloning the BRCA1 cDNA into artificially engineered HindIII and NotI sites in pcDNA3 (27). The vector was a gift of Dr. L. C. Brody. To abolish BRCA1 expression in MCF7, MCF10A, and HB2 cells, shRNA interference was employed using lentivirus vector pGIPZ encoding BRCA1 shRNA or empty vector.

### Western Immunoblots

Cells were plated at a density of 1–2 × 10^6^ cells per 10-cm plate. The next day cells were exposed to treatments as indicated in the legends to figures. After 24–72 h, cells were collected by scraping, washed with ice-cold phosphate-buffered saline (PBS), and lysed with RIPA buffer (150 mM NaCl, 1% NP-40, 0.5% deoxycholic acid, 0.1% SDS, 0.5 M Tris pH 8), supplemented with complete mini-protease inhibitor cocktail (Roche Diagnostics GmbH, Mannheim, Germany). Protein concentration was determined with the Pierce BCA protein assay kit (Thermo Scientific, Rockford, IL, USA). Samples (50 μg) were resolved on 6 and 10% SDS-PAGE, transferred to Protran BA-83 0.2 μm nitrocellulose membrane (Whatman, Piscataway, NJ, USA), blocked with 5% skim milk and immunoblotted with antibodies against phospho-IGF1R (Cat. 3024, directed against Tyr1135/1136), total-IGF1R β-subunit (Cat. 3027), phospho-AKT (Cat. 9271, against Ser473), total-AKT (Cat. 9272), phospho-ERK1/2 (Cat. 9106, against Thr202/Tyr204), and BRCA1 (Cat. 9010). Antibodies were obtained from Cell Signaling Technology (Beverly, MA, USA). Antibodies against total-ERK1/2 (Cat. K23), actin (I-19; sc-1616), and Cbl (C-15; sc-170) were obtained from Santa Cruz Biotechnology (Santa Cruz, CA, USA). The membranes were washed, incubated with the corresponding horseradish peroxidase-conjugated secondary antibody, probed with EZ-ECL enhanced chemiluminescence detection kit (Biological Industries, Beit-Ha-Emek, Israel), and then exposed to Fuji Super RX film (Tokyo, Japan). The expression of β-actin, tubulin, or Cbl was measured as a loading control.

### Immunofluorescence Studies

HCC1937, MCF7, MCF10A, and HB2 cells were plated in 24-well plates on glass cover slips at a density of 1 × 10^4^ cells/well. The cells were serum starved for 24 h, after which the medium was changed to serum-free media with or without MK-0646 antibody (10 μg/ml). After an additional 24 h, the cells were fixed with 4% paraformaldehyde, permeabilized, and stained with DAPI (blue) and anti-IGF1R/Alexa555-goat anti-rabbit antibody (red). Immunofluorescence was visualized by confocal microscopy.

### RT-qPCR

Cells were plated at a density 1 × 10^6^ cells per 10-cm plate. After 24 h, cells were collected by scrapping and washed with ice-cold PBS. Total RNA was isolated using an RNeasy Mini Kit (Qiagen GmbH, Hilden, Germany). RNA concentration and quality were determined by optic density measurement (260 and 280 nm). The quality of the samples was further verified by electrophoresis on 1% agarose gels and stained with ethidium bromide to visualize the 18S and 28S rRNA bands. Complementary (cDNA) was prepared using random primers and a High Capacity cDNA Reverse Transcription Kit (Applied Biosystems, Foster City, CA, USA). cDNA was subjected to RT-qPCR on a StepOnePlus Real Time PCR System using a Power SYBR Green PCR Master Mix (Applied Biosystems). RT-qPCR was performed according to the manufacturer’s instructions using the following primer sets: BRCA1 forward, 5′-TTTATCTGCTCTTCGCGTTGAA-3′; reverse, 5′-TCAACTCCAAGACAGATGGGACA-3′; IGF1R forward, 5′-AAGCTCTATCGAGTCGAGTACG-3′; reverse, 5-GAAGCTCAGAGAACCCATCC-3; actin forward, 5′-TGGACCTCATGGCCCACA-3′; reverse, 5-TCAAGGGGTCTACATGGCAA-3. The number of PCR cycles to reach the fluorescence threshold was the cycle threshold (*C*_t_). Each cDNA sample was tested in triplicate using actin as a negative control, and mean *C*_t_ values are reported. For each reaction, a “no template” sample was included as a negative control. The relative expression of each sample was calculated using the 2^−(ΔΔ*C_t_*)^ method. Results are shown as fold-changes relative to controls.

### Proliferation Assays

Cells were plated in triplicate in 96-well plates (MCF10A, 6 × 10^3^ cells/well; MCF7 and HB2, 3 × 10^3^ cells/well; HCC1937, 4 × 10^3^ cells/well) and allowed to attach overnight. The medium was replaced with fresh treatment-containing medium and the cells were propagated for an additional 72 h. Cell viability was determined by an XTT cell proliferation kit (Biological Industries) by replacing the medium with fresh medium containing charcoal-stripped FBS (in order to prevent interference of treatment color with XTT signal), and the addition of XTT for 2–3 h according to the manufacturer’s instructions. The resulting signal was measured by a Power Wave X 340-I ELISA reader (Biotek Instruments, Winooski, VT, USA) in at least three independent assays.

### Cell Cycle Analyses

MCF7 and HCC1937 cells were seeded onto 6-well plates (0.5 × 10^6^ cells/well) for 24 h. Cells were then serum starved for an additional 24 h and incubated in the presence or absence of IGF1 with or without MK-0646 for 24 h. After incubation, cells were washed with PBS, trypsinized, centrifuged, resuspended in citrate buffer, and stored at −80°C prior to analysis. The cells were thawed and permeabilized before adding propidium iodide. Stained cells were analyzed using a FacsCalibur system (Cytek Development Inc., Fremont, CA, USA).

### Statistical Analyses

The statistical significance of differences was assessed by Student’s *t*-test (two samples, equal variance). Results are presented as mean ± SEM of three independent experiments, performed in triplicate dishes. A *p*-value of 0.05 was considered statistically significant.

## Results

**Figure 1:**
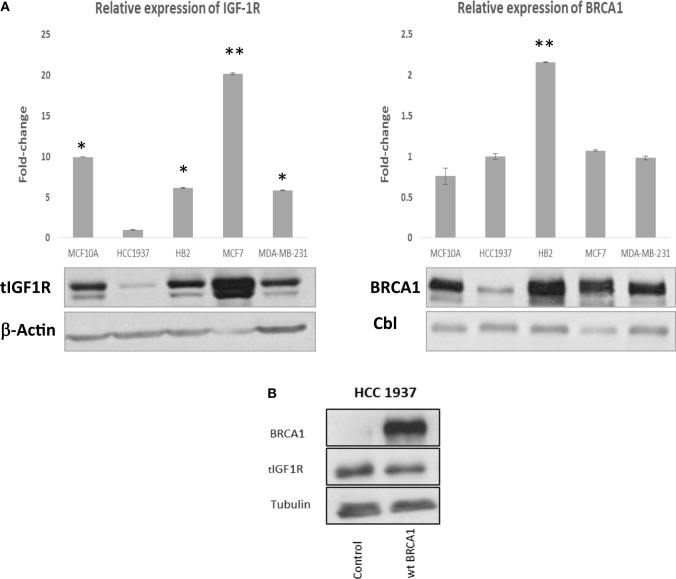
Insulin-like growth factor-1 receptor (IGF1R) gene expression in wild-type- and mutant-BRCA1-containing breast cancer cells. **(A)** Confluent cultures of wild-type BRCA1-expressing MCF7, MCF10A, HB2, and MDA-MB-231, and mutant BRCA1-expressing HCC1937 cells, were harvested and total protein and RNA was extracted. The bar graphs represent the IGF1R and BRCA1 mRNA levels in the various cell lines, as measured by RT-qPCR. An arbitrary value of 1 in the *y*-axis was given to the mRNA levels in HCC1937 cells. Bars represent mean ± SEM of three independent experiments (*p < 0.05 versus HCC1937; **p < 0.01 versus HCC1937). Equal amounts of protein (50 μg) were separated by 6 and 10% SDS-PAGE, transferred to nitrocellulose filters and blotted with anti-BRCA1 or anti-total IGF1R antibodies, respectively. The positions of the ~220-kDa BRCA1, ~97-kDa IGF1R β-subunit, 42-kDa β-actin, and 100-kDa Cbl bands are indicated. **(B)** Effect of BRCA1 expression on endogenous IGF1R levels. HCC1937 cells were seeded in 10-cm plates at a density of 1 × 10^6^ cells per plate. After 24 h, cells were transiently transfected with 10 μg of the pcDNA3-BRCA1 expression vector, or empty vector, using the jetPRIME reagent. After 48 h, cells were harvested, and levels of BRCA1 and endogenous IGF1R were assessed by Western blotting. Tubulin was used as a loading control.

The *IGF1R* gene has been identified as a downstream target for BRCA1 action (22). Wild-type, but not mutant, BRCA1 inhibited *IGF1R* promoter activity, leading to reduced IGF1R biosynthesis and, potentially, diminished mitogenic activity (20). Given the differential regulation of *IGF1R* expression by wild-type and mutant BRCA1, we examined in the present study the hypothesis that BRCA1 status may impinge upon the effectiveness of IGF1R-directed target therapies. In initial experiments, we measured endogenous BRCA1 and IGF1R levels in a number of breast cancer cell lines expressing a wild-type or a mutant *BRCA1* gene. MCF7 cells, containing a wild-type BRCA1, expressed higher levels of BRCA1 protein than HCC1937 cells, which express a mutant BRCA1 (Figure 1A, right panel). Of interest, BRCA1 mRNA levels in both cell lines were comparable. Enhanced BRCA1 protein levels were also detected in additional breast cancer cell lines including a wild-type BRCA1 (i.e., MCF10A, HB2, and MDA-MB-231). Despite the reported inhibition of *IGF1R* gene expression by wild-type BRCA1, basal IGF1R levels were significantly lower in HCC1937 than in MCF7 cells (Figure 1A, left panel). This finding, most probably, reflects the fact that multiple transcription factors are involved in *IGF1R* gene *trans*-activation and repression.

To directly investigate the ability of BRCA1 to regulate IGF1R levels, HCC1937-derived clones overexpressing wild-type BRCA1 were generated. Since a transient expression vector was used, BRCA1 expression was monitored daily for 2 weeks. BRCA1 mRNA levels were high during the first 3 days after transfection and then gradually decreased (data not shown). Western blot analysis revealed a slight (15–20%) reduction in endogenous IGF1R levels in BRCA1-expressing clones (Figure 1B). This result replicates previous reports showing that the *IGF1R* gene constitutes a downstream target for inhibitory regulation by BRCA1 (20, 21).

**Figure 2:**
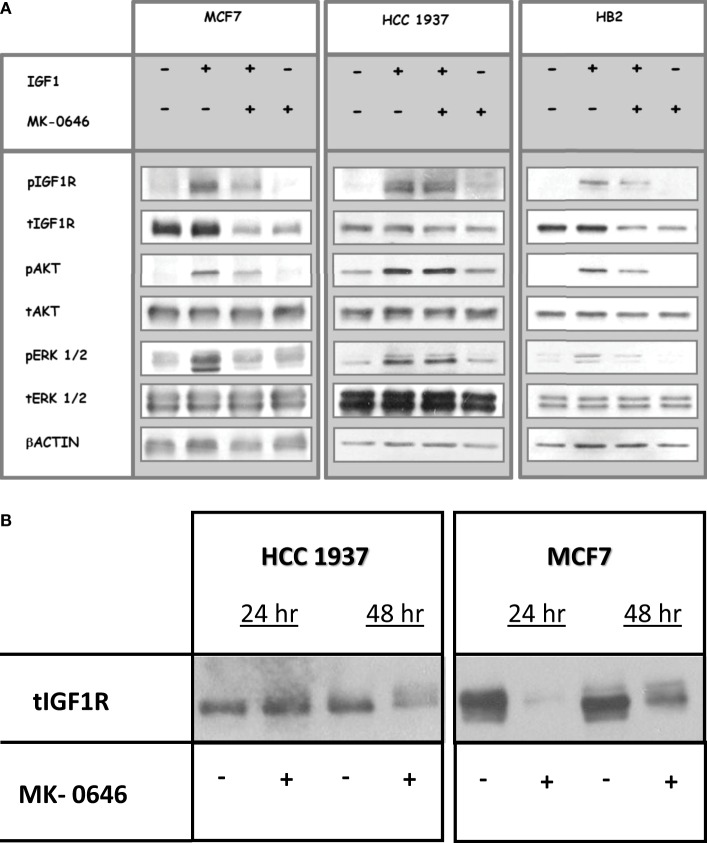
Effect of MK-0646 treatment on the insulin-like growth factor-1 receptor (IGF1R) signaling pathway. **(A)** MCF7, HCC1937, and HB2 cells were seeded in 10-cm plates at a density of 2 × 10^6^ cells per plate. After 24 h, cells were treated with MK-0646 (10 μg/ml) for 5 h, followed by insulin-like growth factor-1 (IGF1) (50 ng/ml) treatment during the last 10 min. At the end of the incubation period, cells were lysed, and the levels of phospho- and total IGF1R, AKT, and ERK1/2 were measured by Western blot analysis. Equal loading was confirmed by β-actin measurement. The autoradiogram shows results of a typical experiment, repeated at least three times with similar results. **(B)** Effect of long-term MK-0646 treatment on endogenous IGF1R levels. MCF7 and HCC1937 cells were seeded in 10-cm plates at a density of 2 × 10^6^ cells per plate for 24 h. Cells were then treated for 24 or 48 h with MK-0646, after which IGF1R levels were measured by Western blots. The figure shows results of an experiment repeated three times with similar results.

To assess the impact of MK-0646, a humanized monoclonal antibody against IGF1R, on IGF1-mediated signaling in cells with different BRCA1 backgrounds, MCF7, HCC1937, and HB2 cells were treated with the antibody for 5 h, in the presence or absence of IGF1 (50 ng/ml) during the last 10 min of the incubation period. Phospho- and total IGF1R, AKT, and ERK1/2 were measured by Western blots (Figure 2A). As expected, IGF1-stimulated IGF1R, AKT and ERK1/2 phosphorylation in all of the cell lines (compare lanes 1 versus 2 in each autoradiogram). MK-0646 decreased the IGF1-stimulated IGF1R phosphorylation in wild-type BRCA1-expressing MCF7 and HB2, but not in mutant BRCA1-expressing HCC1937, cells (compare lanes 2 versus 3). Likewise, the activation of downstream mediators AKT and ERK1/2 was attenuated by MK-0646 antibody in MCF7 and HB2, but not in HCC1937, cells.

Next, we evaluated the ability of long-term MK-0646 antibody treatment to inhibit IGF1R expression in breast cancer cells expressing a wild-type or a mutant BRCA1. As shown in Figure 2B, MK-0646 largely reduced IGF1R levels in MCF7 cells at both 24 and 48 h. This reduction in IGF1R levels may explain the attenuation in IGF1R activation seen in wild-type BRCA1-expressing cells. In contrast, a relatively minor MK-0646-induced IGF1R reduction was noticed in BRCA1 mutant HCC1937 cells after 48 h, but not 24 h, treatment.

**Figure 3:**
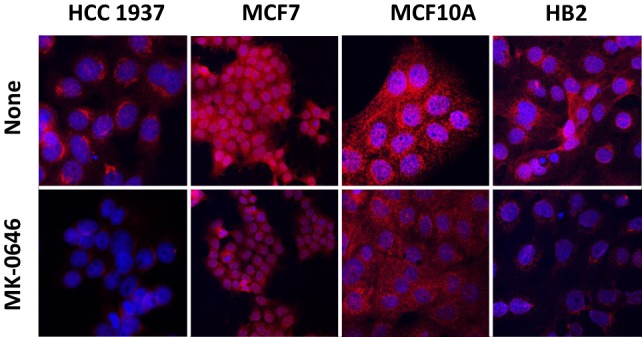
Effect of MK-0646 on insulin-like growth factor-1 receptor cellular localization. HCC1937, MCF7, MCF10A, and HB2 cells were plated in 24-well plates on glass cover slips at a density of 1 × 10^4^ cells/well. The cells were serum starved for 24 h, after which the medium was changed to serum-free media, including or lacking MK-0646 antibody (10 μg/ml). After 24 h, the cells were fixed with 4% paraformaldehyde, permeabilized, and stained with anti-IGF1R/Alexa555-goat anti-rabbit antibody (red) and DAPI (blue).

In previous studies, we provided evidence that IGF1R may undergo nuclear translocation in breast cancer cells (28, 29). To evaluate the impact of BRCA1 status on the subcellular distribution of IGF1R, HCC1937, MCF7, MCF10A, and HB2 cells were stained with a specific IGF1R antibody and for DNA with DAPI. Merged pictures showed that IGF1R was predominantly cytoplasmic but was also detectable in the nuclei of wild-type BRCA1-containing cells (Figure 3). In mutant BRCA1-expressing HCC1937 cells, on the other hand, IGF1R staining was mainly perinuclear. MK-0646 treatment caused a reduction in the intensity of the IGF1R staining in all of the cell lines.

**Figure 4:**
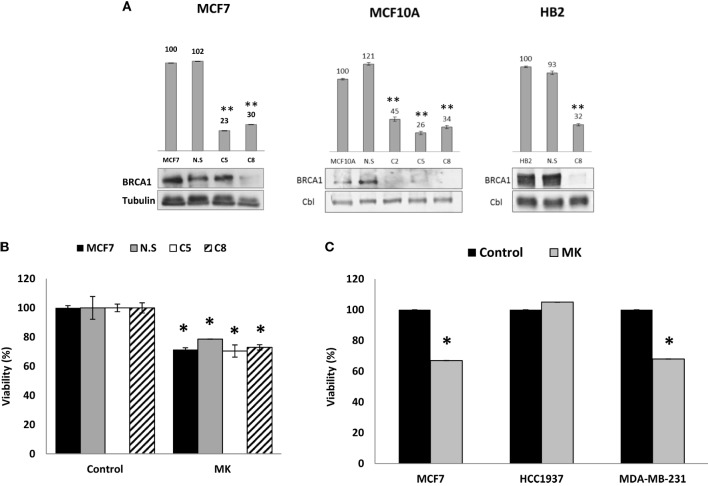
Effect of BRCA1 silencing on the anti-proliferative activity of MK-0646. **(A)** MCF7, MCF10A, and HB2 cells were seeded in 10-cm plates at a density of 1 × 10^6^ cells per plate. After 24 h, cells were infected with 3 μg of the lentivirus vector pGIPZ encoding BRCA1 shRNA or empty vector (NS). Levels of BRCA1 mRNA and protein in untransfected cells, empty vector-transfected cells, and a number of selected clones were measured by RT-qPCR (bar graphs) and Western blots, respectively. Figures above the bars denote arbitrary units of absorbance. Equal loading was confirmed by Cbl or tubulin measurement. Bars represent mean ± SEM of three independent experiments (**p < 0.01 versus respective control). **(B)** Untransfected MCF7 cells, empty vector-transfected cells (NS), and MCF7-BRCA1 knockdown cells (C5 and C8 clones) were plated in 96-well plates at a density of 3 × 10^3^ cells/well. The cells were serum starved for 24 h, after which the medium was changed to serum-free media, including or lacking MK-0646 antibody (MK, 10 μg/ml). Cell proliferation was examined by XTT assays. Bars represent mean ± SEM of three independent experiments (*p < 0.05 versus respective control). **(C)** Wild-type BRCA1-containing MCF7 and MDA-MB-231 cells, and mutant BRCA1-containing HCC1937 cells, were seeded in 96-well plates at a density of 3 × 10^3^ cells/well. The effect of MK-0646 on cell proliferation was assessed as described above. Bars represent mean ± SEM of three independent experiments (*p < 0.05 versus control).

Given the different responses to MK-0646 inhibitor treatment between wild-type BRCA1-expressing MCF7 and HB2 cells, on one hand, and mutant BRCA1-containing HCC1937 cells, on the other hand, and in view of the different levels of BRCA1 protein, we next investigated whether reduced levels of wild-type BRCA1 might affect the response to MK-0646 in terms of proliferation. To this end, MCF7-derived clones with a silenced BRCA1 were generated using shRNA interference with a lentivirus vector. As shown in Figure 4A, this construct effectively inhibited BRCA1 mRNA and protein expression in MCF7, MCF10A, and HB2 cell lines. MCF7 cells (untransfected and empty vector-transfected) and MCF7-BRCA1 knockdown cells (C5 and C8 clones) were treated with MK-0646 antibody for 48 h, and cell viability was determined by XTT assays. Results obtained demonstrate that viability was similar in untransfected MCF7 cells (solid bars) and empty vector-transfected MCF7 cells (gray bars), compared to MCF7-BRCA1 knockdown clones (open and dashed bars). Addition of MK-0646 caused a 20–30% decrease in proliferation rates compared to controls in all of the cell lines (Figure 4B). These results demonstrate that MK-0646 antibody inhibited proliferation despite a major decrease in levels of wild-type BRCA1. Similarly to MCF7 cells, MK-0646 inhibited proliferation in wild-type BRCA1-expressing MDA-MB-231, but not in mutant BRCA1-expressing HCC1937, cells (Figure 4C).

**Figure 5:**
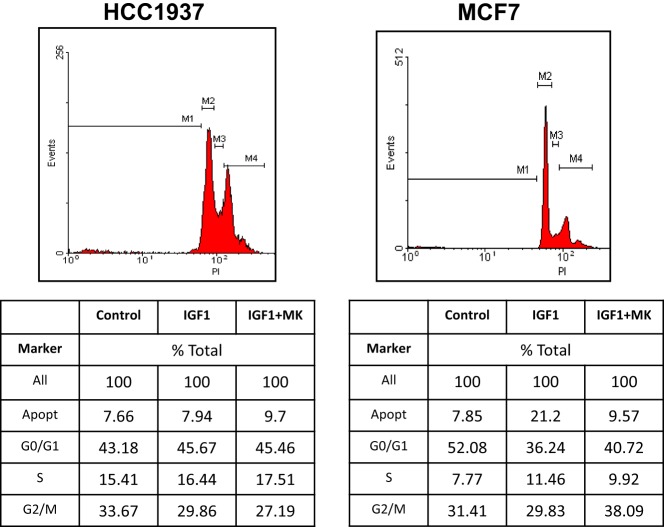
Effect of MK-0646 on the cell cycle in MCF7 and HCC1937 cells. MCF7 and HCC1937 cells were seeded in 6-well plates at a density of 0.5 × 10^6^ cells per plate. After 24 h cells were serum starved for an additional 24 h, after which they were treated with insulin-like growth factor-1 (IGF1) (50 ng/ml) in the absence or presence of the MK-0646 inhibitor (10 μg/ml). At the end of the incubation period, cells were collected by trypsinization into their own medium to prevent loss of dead cells. Cells were fixed with 70% ice-cold ethanol and stained with propidium iodide. Cell cycle distribution and apoptosis analyses were performed using a FacsCalibur system.

To establish whether the different proliferative responses to MK-0646 treatment in MCF7 and HCC1937 cells were associated with corresponding changes in cell cycle progression, experiments were next performed to characterize the effect of the inhibitor on the cell cycle. To this end, MCF7 and HCC1937 cells were treated with IGF1 with or without MK-0646, after which flow cytometry was performed on propidium iodide-stained cells. In MCF7 cells, IGF1 increased the proportion of cells at the S phase from 7.77 to 11.46% and, concomitantly, decreased the portion of cells at G0/G1 from 52.08 to 36.24% (Figure 5). MK-0646 treatment abrogated the stimulatory effect of IGF1 in MCF7 cells. In contrast, antibody MK-0646 had a negligible effect in mutant BRCA1-expressing HCC1937 cells. In summary, data indicate that the MK-0646 inhibitor was able to abolish the effect of IGF1 on cell cycle progression in wild-type BRCA1-containing MCF7, but not in mutant BRCA1-expressing HCC1937, cells.

**Figure 6:**
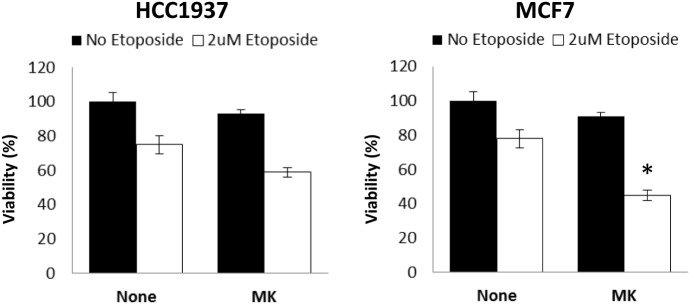
Effect of BRCA1 status on the synergistic activity of MK-0646. MCF7 and HCC1937 cells were plated in 12-well plates at a density of 25 × 10^3^ cells/well. After 24 h, the medium was changed to 5% charcoal-treated fetal bovine serum, including or lacking MK-0646 (MK, 10 μg/ml) with (open bars) or without (solid bars) etoposide (2 μM). Control cells were incubated for the same period of time in the absence of the antibody. Cell proliferation was examined by XTT assays. A value of 100% was given to the viability of untreated cells. Bars represent mean ± SEM of three independent experiments (*p < 0.05 versus no etoposide).

Finally, clinical studies have reported low response rates for single agent IGF1R specific inhibitors. In light of these findings, we tested the response of wild-type and mutant BRCA1-expressing cells to combined treatment with etoposide, a chemotherapeutic agent. As shown in Figure 6, HCC1937 cells showed no significant response to MK-0646, without (solid bars) or with (open bars) etoposide. In contrast, a small but synergistic effect of combined MK-0646 and etoposide treatment was observed in MCF7 cells (55% viability reduction for combined treatment compared to 20% reduction for single agent treatment). Importantly, the synergy was observed in non-stimulated cells, pointing toward possible clinical significance.

## Discussion

The IGF1 hormonal axis and, in particular, the IGF1R have emerged in recent years as promising therapeutic targets in oncology (13, 14, 30, 31). Empirical support to this view was provided by preclinical studies showing that IGF1R *hyper*activation constitutes a fundamental prerequisite for cancer development (7). However, the vast majority of Phase III studies in unselected patients using IGF1R monoclonal antibodies have been disappointing (30). As a result of these negative outcomes, there is an urgent need to identify predictive biomarkers that may identify potential responders. Despite the reported interactions between the IGF1 and BRCA1 signaling pathways, the impact of BRCA1/2 mutational status on selective IGF1R-targeted therapies has not yet been addressed (22, 32).

The data presented here provide evidence that an intact BRCA1 signaling pathway is required for efficient IGF1R-directed targeting. In terms of IGF1R signaling pathway activation, IGF1-stimulated IGF1R, AKT and ERK1/2 phosphorylation in all of the cell lines investigated. However, whereas short-term MK-0646 treatment prevented phosphorylation of IGF1R and downstream mediators in wild-type BRCA1-expressing MCF7 and HB2 cells, it was unable to prevent activation in mutant BRCA1-containing HCC1937, cells. In addition, MK-0646 was able to lessen proliferation of MCF7 and MDA-MB-231, but not of HCC1937, cells. These data are consistent with inability of the blocking antibody to operate in breast cancer cells with a disrupted BRCA1 gene. Alternatively, it might be conceivable that the antibody requires relatively high basal IGF1R levels in order to elicit its inhibitory action. Consistent with this notion, HCC1937 cells express relatively low levels of IGF1R. Results of prolonged (24 and 48 h) exposures to MK-0646 indicate that the antibody reduced IGF1R protein expression in MCF7 cells, whereas a modest reduction was seen at 48 h in mutant BRCA1-containing HCC1937 cells.

Of interest, differences in IGF1R cellular distribution were seen between cells expressing wild-type or mutant BRCA1. Thus, whereas IGF1R was predominantly cytoplasmic, with a noticeable nuclear presence, in wild-type BRCA1-containing cells, IGF1R staining was mainly perinuclear in mutant BRCA1-expressing HCC1937 cells. While the biological role of nuclear IGF1R is still unclear, the finding that the receptor does not translocate to nucleus in cells with a mutant BRCA1 gene may identify BRCA1 as a potential player in nuclear translocation (29).

The interplay between the IGF1 and BRCA1 cellular pathways is very complex. Wild-type BRCA1 was shown to inhibit *IGF1R* gene transcription by repressing promoter activity (20). Conversely, a truncated form of BRCA1 (185delAG, a mutation with high frequency among Ashkenazi Jews) displayed a reduced activity. Electrophoretic mobility shift assays using the *in vitro* translated BRCA1 protein revealed no BRCA1 binding to the *IGF1R* promoter sequence (21). Coherent with the failure of mutant BRCA1 to suppress *IGF1R* gene transcription, immunohistochemical analyses of primary breast tumors derived from a cohort of 185delAG BRCA1 mutation carrier patients revealed almost twofold higher IGF1R levels than in sporadic breast tumors (24). Of clinical relevance, *loss-of-function* mutation of tumor suppressor p53 in human cancer may affect the capacity of BRCA1 to inhibit the *IGF1R* gene (33). Specifically, BRCA1 was capable of inhibiting *IGF1R* promoter activity in p53-expressing and p53-null backgrounds, but not in mutant p53-containing cells. Therefore, the mutational status of p53 is a critical determinant in IGF1R targeting.

Interactions between the IGF1 and BRCA1 signaling pathways are not restricted to breast cancer. We have previously demonstrated high BRCA1 levels in prostate cancer in comparison to normal prostate tissue (34). In addition, an inverse correlation between BRCA1 and IGF1R levels was seen in androgen receptor (AR) negative prostate cancer cell lines. Coexpression experiments revealed that BRCA1 inhibited *IGF1R* promoter activity in AR negative cells while stimulating promoter activity in AR positive cells. Hence, data indicate that the mechanism of action of BRCA1 involves modulation of the *IGF1R* gene in a cell-specific manner. Finally, BRCA1 was also shown to inhibit IGF1R expression in uterine serous carcinoma cells (35).

Activation of BRCA1 following DNA damage, oxidative stress, or other cellular insults may lead to a reduction in IGF1R levels and IGF1 action. As a result of this negative control cells remain at a post-mitotic state and out of the cell cycle. *Loss-of-function* mutation of BRCA1 in familial cancer may abolish its inhibitory role, leading to constitutive *hyper*activation of the *IGF1R* gene, a typical hallmark of cancer cells (22). The existence of a bidirectional regulatory loop between the IGF1 and BRCA1 signaling pathways was suggested by studies showing that IGF1 and IGF2 enhance BRCA1 gene expression in a dose-dependent manner (23). Data presented here indicate that these complex interactions impinge upon the ability of selective IGF1R inhibitors to block the IGF1 pathway for therapeutic purposes. The mutational status of BRCA1 must be taken into account when selecting patients for IGF1R targeting protocols.

In summary, identification of molecular predictors of sensitivity to IGF1R inhibitors constitutes a critical field of research in oncology. Given the modest benefit achieved with current therapeutic approaches, discovery of novel biomarkers is expected to have major translational implications. Extensive molecular profiling revealed that a number of components of the IGF pathway, including IRS2 and IGFBP5, may play key roles in determining the sensitivity of cancer cells to humanized IGF1R antibody figitumumab (36). Similarly, IGF1R expression levels and activation status, as well as additional downstream mediators, might also help selecting patients for targeting therapy (4, 7, 13). In conclusion, our study identifies BRCA1 as a novel potential biomarker in breast cancer. Future studies will address this paradigm in the clinical setting.

## Author Contributions

TC-S, RB, and HW conceived of and designed the experiments. The experimental procedures were performed by TC-S and ZC and were analyzed by TC-S, RB, ZC, and HW. TC-S and HW prepared the manuscript.

## Conflict of Interest Statement

The authors declare that there is no conflict of interest that could be perceived as prejudicing the impartiality of the research reported.
